# Referral trajectories in patients with vertigo, dizziness and balance disorders and their impact on health-related quality of life and functioning: results from the longitudinal multicenter study MobilE-TRA

**DOI:** 10.1007/s00415-022-11060-8

**Published:** 2022-03-30

**Authors:** Benedict Katzenberger, Daniela Koller, Ralf Strobl, Rebecca Kisch, Linda Sanftenberg, Karen Voigt, Eva Grill

**Affiliations:** 1 Institute for Medical Information Processing, Biometry and Epidemiology, Ludwig-Maximilians-Universität München, Marchioninistraße 15, 81377 Munich, Germany; 2 Munich Center of Health Sciences, Ludwig-Maximilians-Universität München, Munich, Germany; 3 German Center for Vertigo and Balance Disorders, University Hospital, Ludwig-Maximilians-Universität München, Munich, Germany; 4 Institute of General Practice and Family Medicine, University Hospital, Ludwig-Maximilians-Universität München, Munich, Germany; 5 Department of General Practice/Medical Clinic III, Faculty of Medicine, Technische Universität Dresden, Dresden, Germany

**Keywords:** Referral trajectories, Vertigo, dizziness and balance disorders, State sequence analysis, Health-related quality of life, Functioning, MobilE-TRA

## Abstract

**Background:**

Due to reported barriers in the management of patients with vertigo, dizziness and balance problems (VDB), referral trajectories starting from primary care might be determined by other factors than medical necessity. The objective of this paper was to examine the impact of disease-related and other determinants on referral trajectories of older patients with VDB and to investigate, how these trajectories affect the patients’ functioning and health-related quality of life (HRQoL).

**Methods:**

Data originate from the longitudinal multicenter study MobilE-TRA, conducted in two German federal states. Referrals to neurologists or ear-nose-throat (ENT) specialists were considered. Referral patterns were visualized using a state sequence analysis. Predictors of referral trajectories were examined using a multinomial logistic regression model. Linear mixed models were calculated to assess the impact of referral patterns on the patients’ HRQoL and functioning.

**Results:**

We identified three patterns of referral trajectories: primary care physician (PCP) only, PCP and neurologist, and PCP and ENT. Chances of referral to a neurologist were higher for patients with a neurological comorbidity (OR = 3.22, 95%-CI [1.003; 10.327]) and lower for patients from Saxony (OR = 0.08, 95%-CI [0.013; 0.419]). Patients with a PCP and neurologist referral pattern had a lower HRQoL and lower functioning at baseline assessment. Patients with unspecific diagnoses also had lower functioning.

**Conclusion:**

Referral trajectories were determined by present comorbidities and the regional healthcare characteristics. Referral trajectories affected patients’ HRQoL. Unspecific VDB diagnoses seem to increase the risk of ineffective management and consequently impaired functioning.

**Supplementary Information:**

The online version contains supplementary material available at 10.1007/s00415-022-11060-8.

## Introduction

With an annual prevalence of 9% in medical claims databases [1] and a total of up to 4.2% of all visits, vertigo, dizziness and balance problems (VDB) are among the most frequent reasons for older adults to consult primary care [2].

There is evidence for inappropriate management of VDB in primary care, both in Germany and internationally [3, 4]. The reasons for this are still poorly understood.

Usually, patients with VDB are initially seen by primary care physicians (PCP), who then have to decide whether additional tests, a strategy of watchful waiting, referral to secondary care, or straightforward therapy are needed. This decision is additionally challenging since it has been shown that dizziness in the aged can have multiple causes [5, 6]. Distinct treatable vestibular disease entities, dizziness caused by medication, cardiovascular disease or diabetes may align with symptoms of the ageing of vestibular, proprioceptive or somatosensory systems. Therefore, a considerable percentage of older adults with confirmed vestibular vertigo experience limited functioning and impairment in their health-related quality of life (HRQoL) due to their untreated VDB [7, 8].

Ideally, referral trajectories from the PCP to a specialist should be guided by the underlying cause, making use of the specialist’s expertise for the respective disease [9, 10]. However, PCPs report barriers in the referral routines of older patients with VDB. This may be due to a lack of experience with specific diagnostic tests, fragmentation of the health care system, not having enough time for interaction with patients, or missing guidance, such as missing management standards for VDB [11]. Thus, it seems hardly surprising that many patients consult multiple health care professionals, often without getting a definite diagnosis [3]. To give an example, in the US over 36% of older patients with VDB were seen by more than three health professionals, yet 40% of the patients with VDB remained without definite diagnosis [4].

As a result, referral trajectories in VDB might be determined not only by medical necessity but also by other factors. To date, very little is known about the role of such determinants of referral in patients with VDB. Taking a more generalized look at other indications reveals, that such other determinants for referral decisions from primary care to a specialist can in general be categorized into three dimensions: the characteristics of the patients, the characteristics of the PCP, and the surrounding health care characteristics [12–15].

Regarding patient characteristics, male gender and advanced age [12] as well as a higher educational level [13] increase the likelihood for referral. Chances of referral further differed based on the experience of the PCP [15]. Regarding system characteristics, studies conducted in the US and England have shown that differences in the local health care characteristics did influence referral frequency [12, 14].

It has been shown that patients with VDB under usual care conditions often were not able to improve in patient-relevant outcomes [16], yet that improvement is possible when the causes of VDB are adequately cared for [17]. Referral trajectories may play an important role in whether or not such an improvement can be obtained.

The objective of this paper thus was to investigate the impact of both disease-related and not disease-related determinants on referral trajectories in patients with VDB. Also, we wanted to investigate, how current referral trajectories affect patient-relevant outcomes such as functioning and HRQoL.

## Methods

### Study design, study population, and data collection procedures

Data for this research project emanated from the longitudinal multicenter study MobilE-TRA conducted in two German federal states (Bavaria and Saxony) from September 2017 until October 2019. A more detailed description of the study can be found elsewhere [18]. In brief, patients aged 65 years and older were included if they had consulted their PCP for an acute episode of VDB in the last quarter. The identification of suitable individuals was accomplished by approaching PCPs who were willing to participate and asking them to search their patient databases for the following ICD-10 codes associated with VDB: R42, A88.1, E53.8, F45.8, G11.8, G43.1, G45.0, G62, G63, H55, H83.0–2, I95.1, and N95.1. A detailed list of the ICD-10 codes and the related diagnoses are listed in Online resource 1. Patients additionally had to have statutory health insurance (covering approx. 90% of the German population [19]) as well as sufficient command of the German language.

Baseline assessment was conducted in between September 2017 and August 2018 and consisted of a self-administered health questionnaire, which was sent to each patient. Participating PCPs were asked to complete an adapted version of the standardized Questionnaire of Chronic Illness Care in Primary Care (QCPC) [20]. PCP in addition were asked to give information on each included patient comprising an additional self-developed baseline questionnaire covering the diagnosis, information on referrals to other specialists, and the treatment strategy. The study consisted of two additional waves: Follow-up invitations were sent to the patients 6 months and 12 months after individual baseline dates. Assessments for follow-up one and follow-up two consisted of the self-administered health questionnaire, only.

Ethics approval for MobiLE-TRA was obtained from the Ethics Committee of the Ludwig-Maximilians-Universität München (#17-443) and the Ethics Committee of the Technische Universität Dresden (#E365092017). Written informed consent was obtained from all participants. The study was performed in accordance with the Declaration of Helsinki principles.

The population of this analysis is composed of all patients with VDB with valid information on consulted physicians for baseline and at least one follow-up, based on their questionnaire. A more detailed flowchart can be found in Online Resource 2.

### Referrals

Referrals to specialists were indirectly assessed by asking the patients about the physicians they had been consulting within the last three months prior to each assessment. For this purpose, patients were presented a standardized list of physicians (Questionnaire for Health-Related Resource Use in an Elderly Population—FIMA) [21]. As the most obvious choice of consultation in case of VDB is the PCP, or a referral to a neurology or otorhinolaryngology specialist, we concentrated for our analyses on PCPs, neurologists and ear-nose-throat (ENT) specialists [22].

### Disease-related and other determinants of referral trajectories

VDB diagnosis was reported by the PCP during baseline assessment. To facilitate analysis and in line with current classifications [23], we categorized these diagnoses based on the reported cause of VDB into vestibular vertigo (e.g., BPPV, Meniere's disease, and Vestibular neuritis), central vertigo (e.g., stroke and vestibular migraine), other specific diagnoses (i.e., cardiovascular problems, psychogenic dizziness), and unspecific vertigo. We hypothesized that referrals to a neurologist should be more likely in the case of central vertigo. Vestibular vertigo should increase the chance of referral to an ENT-specialist. All other specific diagnoses should generally not lead to referral to neither a neurologist nor an ENT-specialist. The diagnosis was labeled as ‘not specified’ if the PCP did not specify a diagnosis but enrolled the patient in the VDB survey. For further analyses, VDB diagnosis was also categorized into a binary variable, listing all diagnoses with a specific cause (vestibular vertigo, central vertigo, cardiovascular problems, and psychogenic dizziness) as ‘specific’ VDB, whereas all other cases where no diagnostic decision was made were summarized as ‘unspecific’ VDB. Diagnoses that were labeled as ‘not specified’ were not included into this binary variable and thus were treated as missing values.

Comorbidities were reported by the PCP using the Charlson Comorbidity Index [24]. Following recommendations [25], we added further comorbidities to the index list that had shown to be of high relevance in older adult populations and potentially might influence HRQoL [26]. A detailed list of the comorbidities is shown in the Online Resource 3. These comorbidities were then categorized into ‘neurological’ comorbidities (including stroke), ‘ENT – related’ comorbidities, and ‘none / other’ comorbidities.

Information on gender (male/female) and age was based on patients’ self-report. Education levels were categorized on the basis of the German educational system as follows: no graduation or lower secondary education (equals 9 years of school or less), lower secondary education (equals 10 years of school), upper secondary education (equals 12 or 13 years of school) and tertiary education (university, university of applied sciences). The PCP’s experience was approximated by the number of years that a PCP was working after licensure. Differences in referrals due to differences in the surrounding health care characteristics were addressed by including the federal state as binary variable (Bavaria/ Saxony).

### Health-related quality of life and functioning

HRQoL was measured using the visual analog scale (VAS) which is part of the EuroQol Five-Dimensional Five-Level Questionnaire (EQ-5D-5L), developed by the EuroQol Group [27]. Patients were asked to rate their present health on a scale from 0 to 100, where 100 indicates perfect health and 0 indicates the worst health imaginable.

Functioning was assessed by the two-scale version of the Vestibular Activities and Participation questionnaire (VAP) [28], which is consisting of two separate scales. VAP Scale 1 measures patient-reported functioning regarding activities that are difficult to perform because of their propensity to provoke vertigo or dizziness (activity VAP). VAP Scale 2 indicates immediate consequences of vertigo and dizziness on activities and participation related to mobility (mobility VAP). Interval scaled overall scores (range scale 1 = 0–23; range scale 2 = 0–20) were used with higher scores indicating lower functioning.

### Statistical analysis

#### Constructing referral trajectories and clustering them using state sequence analysis

To identify clusters of similar referrals trajectories, we used state sequence analysis (SSA) which has already been successfully applied in care pathways research settings [29–31].

For the purpose of this study, a referral trajectory is defined as a sequence of distinct combinations of consulted practitioners, technically termed as states, which are ordered in their chronological sequence.

Each individual trajectory consisted of three states—one for each wave. Each trajectory started with the completion of the baseline questionnaire, including the last 6 months prior to that time point, and stopped, when the follow-up two questionnaire was completed. To provide an example: An individual trajectory may consist of PCP consultation at baseline, the simultaneous consultation of the PCP and a neurologist at follow-up one and the sole consultation of the PCP at follow-up 2.

Patients may consult multiple physicians simultaneously. The resulting list of states included in this study therefore is as follows: PCP only, ENT specialist only, neurologist only, PCP and neurologist, PCP and ENT specialist, ENT specialist and neurologist, and PCP, ENT specialist and neurologist. In case of a missing state for baseline assessment, we assumed that every patient did exclusively consult the PCP (as defaulted in our inclusion criteria).

In a second step, dissimilarity between referral trajectories, i.e., the minimal cost to transform one trajectory into another, was measured using optimal matching (OM), based on the transition rates present in the dataset [32].

The clusters of similar referral trajectories finally were obtained using a Partitioning Around Medoids (PAM) – algorithm [33], which was based on the acquired dissimilarity. The optimal number of clusters was determined by two quality criteria: The weighted average silhouette width (ASWw), measuring the overall consistency of the clusters, and the Hubert’s C index (HC), reflecting the gap between the clustering obtained and the best theoretically possible clustering based on the numbers of groups and distances present. Further details about this approach are described elsewhere [29, 33].

State distribution over time is shown with the help of a state distribution plot, which displays the general pattern of states over time on a group level on the x-axis while the cumulative proportion of patients in the different states is presented on the y-axis. The actual trajectories of each cluster are represented using an index plot, with the trajectories shown on the x-axis while the bar height of each trajectory is proportional to the number of observations assigned to each trajectory.

#### Descriptive analysis, cluster comparison and examination of potential determinants of cluster membership

Summary statistics were calculated for the overall sample and separately for each cluster to compare differences in diagnoses, comorbidities, characteristics of the patients and the PCPs, the federal states, as well as baseline HRQoL and functioning. Mean and standard deviation were reported for continuous variables, relative and absolute frequencies were reported for categorical variables.

A multinomial logistic regression model was calculated based on data from the baseline assessment to test for potential determinants of the identified clusters, with the PCP cluster as a reference.

#### Examining the impact of cluster membership on the patient’s health-related quality of life and functioning

We calculated longitudinal linear mixed models with random intercepts and random slopes to assess whether HRQoL and functioning was determined by the clusters of similar referral trajectories. Regression models were separately calculated for the VAS, the activity VAP Scale 1, and the mobility VAP Scale 2. To address differences between the clusters in the development of HRQoL and functioning over time, we introduced interaction terms between the waves and the clusters. We introduced a second interaction term between the waves and the diagnosis of VDB (specific or unspecific) to address potential differences in the development over time between patients with a specific and an unspecific diagnosis of VDB. Random effects for intercept and slope were reported along with the Bayesian information criterion (BIC).

Variable selections was done using directed acyclic graphs (DAGs) (see Online Resource 4) to avoid bias by over-adjustment or collider bias and to arrive at a parsimonious set of variables, the minimal sufficient adjustment set, for estimating the effect of referral clusters on HRQoL and functioning [34]. The resulting minimal adjustment set consisted of VDB diagnosis, present comorbidities, federal state, gender, age, and education.

To facilitate interpretation of intercept estimates, the minimum age of 65 as set by the inclusion criteria was subtracted from age in years for each patient.

All computational analyses were carried out with R Studio Version 4.0.3 [35] using the TraMineR, Weighted Cluster and nlme libraries [32, 33, 36]. Significance level was set to 5% for all tests conducted. To construct the DAGs for this study, we used DAGitty, a browser-based environment for creating, editing, and analyzing causal models [37].

## Results

### Study population

A total of 19 PCPs (7 from Bavaria, 12 from Saxony; mean age = 54 years; 29% female) recruited 158 patients with VDB. Of these, a total of 141 patients (mean age = 76.8 years, 70% female, 60% from Bavaria) had information on consultations for baseline and at least 1 follow-up and thus were included into the analysis.

A total of 39% had a specific VDB diagnosis (9.2% vestibular vertigo, 12.1% neurological central vertigo, 17.7% other specific diagnoses), 44% of the patients had an unspecific VDB diagnosis.

 Mean baseline HRQoL was 64.2 (SD = 19.9), mean activities VAP was 7.2 (SD = 4.1), and mean mobility VAP was 6.4 (SD = 4.8).

### Clusters of similar referral trajectories

**Fig. 1:**
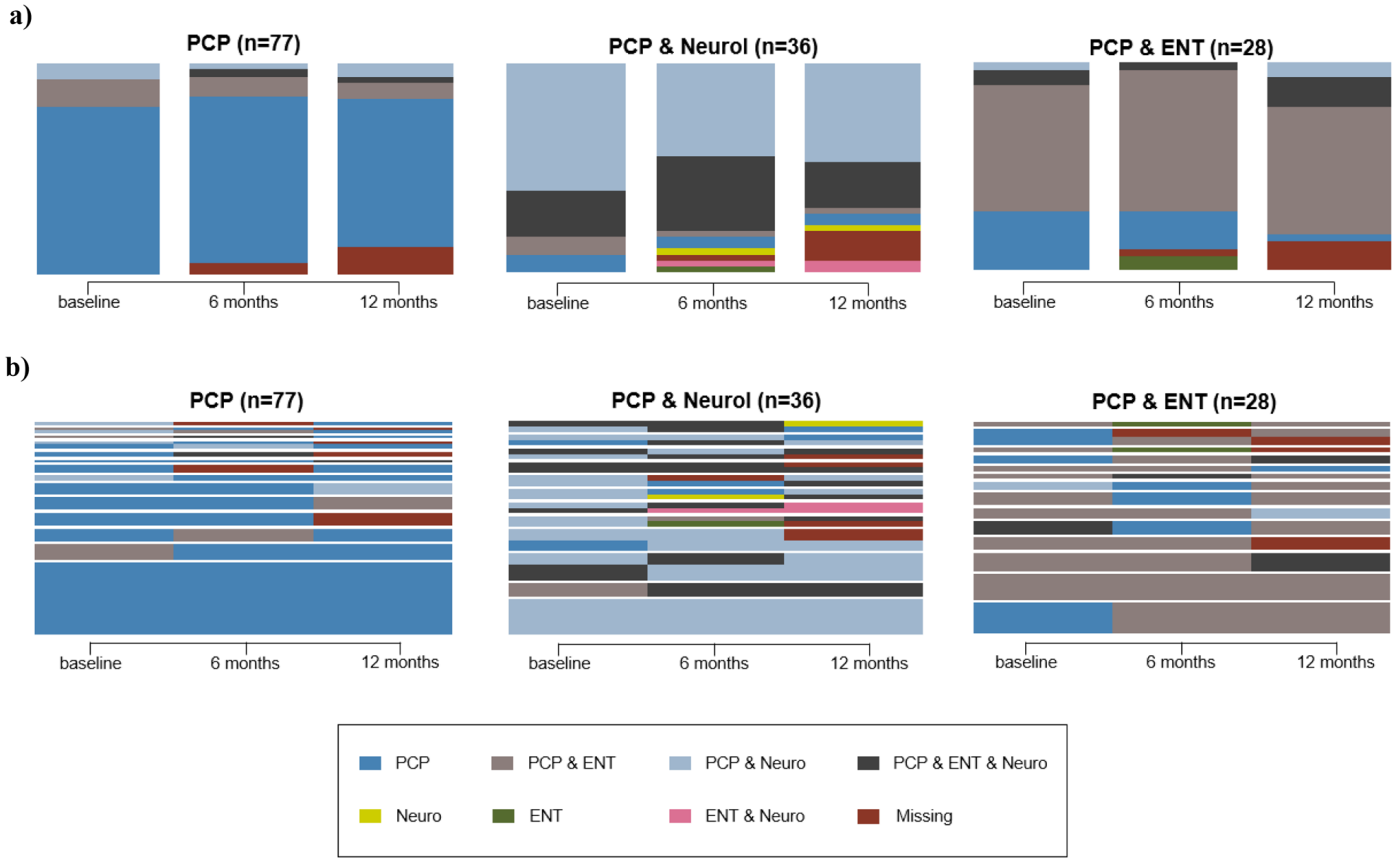
State distribution plot (**a**), displaying the general pattern of states over time on the *x*-axis while presenting the cumulative proportion of patients in the different states on the *y*-axis. Index plot (**b**) representing the actual referral trajectories in each cluster on the *x*-axis. The bar height of each trajectory is proportional to the assigned number of observations

We identified three distinct clusters of similar referral trajectories (see Fig. 1). Cluster 1 (‘PCP’ cluster) consisted of 77 persons that consulted only the PCP and were not or hardly ever referred. Patients in cluster 2 (‘PCP & Neurol’, *n* = 36) most frequently consulted both PCP and neurologists or PCP, Neurologists and ENT simultaneously. Patients in cluster 3 (‘PCP & ENT’, *n* = 28) most commonly consulted both PCP and ENT simultaneously.

### Determinants of cluster membership

Table 1 shows the summary statistics by cluster of similar referral trajectories at baseline assessment.

**Table 1:** Unadjusted summary statistics by trajectory cluster at baseline assessment (*n* = 141)

	Overall	Clusters of similar referral trajectories		
		Cluster ‘PCP’	Cluster ‘PCP & Neurol’	Cluster ‘PCP & ENT’
*N* (%)	141 (100)	77 (55)	36 (25)	28 (20)
Diagnosis of VDB				
Vestibular (*n*, %)	13 (100)	7 (54)	5 (38)	1 (8)
Central (*n*, %)	17 (100)	6 (35)	8 (47)	3 (18)
Unspecific (*n*, %)	62 (100)	34 (55)	16 (26)	12 (19)
Other specific diagnoses (*n*, %)	25 (100)	15 (60)	3 (12)	7 (28)
Not specified (*n*, %)	24 (100)	15 (62)	4 (17)	5 (21)
Comorbidities				
None / Other (*n*, %)	102 (100)	58 (56)	22 (22)	22 (22)
Neurological (*n*, %)	26 (100)	12 (46)	12 (46)	2 (8)
ENT-related (*n*, %)	13 (100)	7 (54)	2 (15)	4 (31)
Location				
Bavaria (*n*, %)	85 (100)	41 (48)	33 (39)	11 (13)
Saxony (*n*, %)	56 (100)	36 (64)	3 (5)	17 (30)
Gender				
Male (*n*, %)	43 (100)	25 (58)	8 (19)	10 (23)
Female (*n*, %)	98 (100)	52 (53)	28 (29)	18 (18)
Age (mean, SD)	76.8 (6.1)	77.2 (5.8)	76.8 (6.5)	76.0 (6.4)
Education^a^				
No graduation (*n*, %)	2 (100)	1 (50)	1 (50)	0 (0)
Lower secondary education 1 (*n*, %)	62 (100)	37 (60)	15 (24)	10 (16)
Lower secondary education 2 (*n*, %)	30 (100)	13 (43)	12 (40)	5 (17)
Upper secondary education (*n*, %)	10 (100)	5 (50)	2 (20)	3 (30)
Tertiary education (*n*, %)	29 (100)	18 (62)	5 (17)	6 (21)
Missing values (*n*, %)	8 (100)	3 (37)	1 (13)	4 (50)
Experience of the PCP (mean, SD)	18.5 (7.4)	18.3 (7.7)	19.1 (5.2)	18.0 (9.3)
Missing values (*n*, %)	6 (100)	4 (66)	1 (17)	1 (17)
HRQoL				
VAS (*mean, SD*)	64.2 (19.9)	68.6 (19.7)	54.3 (20.1)	64.5 (16.4)
Missing values (*n*, %)	4 (100)	2 (50)	2 (50)	
Functioning				
Activity VAP Scale1 (mean, SD)	7.2 (4.1)	6.7 (4.0)	8.3 (4.4)	7.3 (3.9)
Missing values (*n*, %)	51 (100)	29 (57)	15 (29)	7 (14)
Mobility VAP Scale 2 (mean, SD)	6.4 (4.8)	5.9 (4.8)	7.4 (5.2)	6.3 (4.6)
Missing values (*n*, %)	39 (100)	23 (59)	13 (33)	3 (8)

We report mean and SD for continuous variables and absolute and relative frequencies for categorical variables.

*VDB* Vertigo, dizziness and balance problems, *PCP* primary care physician, *Neurol* Neurologist, *ENT* ear, nose and throat, *HRQoL* health-related quality of life, *VAS* Visual analog scale, *VAP* Vestibular Activities and Participation questionnaire, *SD* standard deviation

^a^ Lower secondary education 1 equals 9 years of school, Lower secondary education 1 equals 10 years of school, upper secondary education equals 12 or 13 years of school

Table 2 shows the results of the multinomial logistic regression model, which was computed to test for potential determinants of the identified clusters. Odds ratios (OR) are reported to represent the odds to be in the respective cluster as compared to the odds to be in the reference PCP cluster.

**Table 2:** Multinomial regression models to assess predictors for cluster membership during baseline assessment. The reference cluster is ‘PCP’

	Clusters of similar referral trajectories (Reference cluster is ‘PCP’)	
	Cluster ‘PCP & Neurol’ (OR)	Cluster ‘PCP & ENT’ (OR)
Intercept	6.67 (0.01; 3643.71)	0.55 (0.00; 862.69)
Diagnosis of VDB		
Other specific diagnoses	Reference	Reference
Vestibular	2.49 (0.32; 19.14)	0.36 (0.03; 3.74)
Central	2.81 (0.42; 18.91)	1.34 (0.20; 9.09)
Unspecific	1.35 (0.28; 6.64)	0.64 (0.17; 2.43)
Not specified	1.01 (0.14; 7.34)	0.78 (0.15; 3.97)
Present comorbidities		
None / Other	Reference	Reference
Neurological	**3.22 (1.00; 10.33)**	0.42 (0.08; 2.38)
ENT-related	0.29 (0.03; 2.99)	1.70 (0.37; 7.73)
Location		
Bavaria	Reference	Reference
Saxony	**0.08 (0.01; 0.42)**	2.66 (0.83; 8.57)
Gender		
Male	Reference	Reference
Female	0.96 (0.26; 3.53)	1.23 (0.32; 4.70)
Age	0.96 (0.89; 1.04)	0.99 (0.90; 1.08)
Education^a^		
No graduation or lower secondary education 1	Reference	Reference
Lower secondary education 2	2.17 (0.66; 7.13)	1.34 (0.32; 5.57)
Upper secondary education	1.49 (0.19; 11.63)	1.56 (0.25; 9.64)
Tertiary education	1.47 (0.25; 8.73)	0.78 (0.17; 3.69)
Experience of the PCP	1.01 (0.94; 1.10)	1.00 (0.94; 1.07)
McFadden *R*^2^	0.175	

Odds ratios rounded to two decimals. Significant results are highlighted in bold print

*VDB* Vertigo, dizziness and balance problems, *PCP* primary care physician, *Neurol* Neurologist, *ENT* ear, nose and throat, *OR* Odds ratio

^a^Lower secondary education 1 equals 9 years of school, Lower secondary education 1 equals 10 years of school, upper secondary education equals 12 or 13 years of school

Patients with a neurological comorbidity were significantly more likely to see PCPs and neurologists (OR = 3.22, 95%CI [1.003; 10.327]), as compared to being seen by PCPs exclusively. Patients from Saxony were less frequently referred to neurologists, as expressed by a lower likelihood to be in the PCP & Neurol cluster (OR = 0.08, 95%CI [0.013; 0.419]).

### Examining the impact of referral on the patient’s health-related quality of life and functioning

Adjusted estimates for the association of referral cluster and HRQoL and functioning are shown in Table 3.

**Table 3:** Longitudinal linear mixed models to assess the influence of clusters of similar referral trajectories on health-related quality of life (VAS) and functioning (activity VAP Scale 1 and mobility VAP Scale 2)

	HRQoL	Functioning	
	VAS (95% CI)	Activity VAP Scale 1 (95% CI)	Mobility VAP Scale 2 (95% CI)
Observations (*n*)	318 (109)	195 (89)	231 (99)
Fixed effects			
Intercept	79.83 (66.40; 93.26)	2.96 (-0.34; 6.26)	-1.39 (-5.03; 2.26)
Wave	− 1.49 (− 4.37; 1.39)	**− 1.05 (− 1.78; − 0.32)**	− 0.21 (− 0.92; 0.50)
Cluster of similar referral trajectories			
PCP	Reference	Reference	Reference
PCP & Neurol	**− 12.72 (− 21.27; − 4.17)**	**2.22 (0.01; 4.43)**	1.80 (− 0.70; 4.29)
PCP & ENT	− 7.88 (− 16.95; 1.19)	2.12 (− 0.10; 4.34)	1.70 (− 0.79; 4.19)
Interaction terms cluster of similar referral trajectories * wave			
PCP * wave	Reference	Reference	Reference
PCP & Neurol * wave	**4.04 (0.15; 7.92)**	0.45 (− 0.49; 1.39)	− 0.04 (− 1.02; 0.95)
PCP & ENT * wave	1.76 (− 2.71; 6.24)	0.12 (− 0.87; 1.11)	− 0.43 (− 1.43; 0.56)
Diagnosis of VDB			
Specific	Reference	Reference	Reference
Unspecific	− 6.33 (− 13.02; 0.36)	0.40 (− 1.34; 2.15)	1.65 (− 0.31; 3.60)
Interaction term diagnosis of VDB * wave			
Specific * wave	Reference	Reference	Reference
Unspecific * wave	− 2.11 (− 5.44; 1.22)	**1.16 (0.36; 1.95)**	**0.86 (0.04, 1.67)**
Random effects			
Intercept (SD)	13.80	3.27	4.03
Wave (SD)	3.94	0.56	0.93
BIC	2765.0	1096.1	1331.3

All models are controlled for present comorbidities, the study location, gender, age, and education. Significant results are highlighted in bold print

*PCP* Primary care physician, *VDB* vertigo, dizziness and balance problems, *Neurol* Neurologist, *ENT* ear, nose and throat, *HRQoL* health-related quality of life, *VAS* visual analog scale, *VAP* Vestibular Activities and Participation questionnaire, *CI* confidence interval

Patient-reported HRQoL at baseline was significantly lower for patients in the PCP & Neurol referral cluster (Beta = − 12.72, 95%-CI [− 21.27; − 4.17]). Yet the development of the HRQoL over time, represented by the interaction term of the clusters of similar referral trajectories and the study waves, was significantly better for these patients (4.04, [0.15; 7.92]), resulting in an overall increase over time. Patients in the PCP & Neurol referral cluster had significantly worse functioning at baseline (2.22, [0.01; 4.43]).

Functioning increased over time for patients with a specific VDB diagnosis (− 1.05, [− 1.78; − 0.32]). The development of functioning in patients with an unspecific diagnosis was significantly worse for both the activity VAP Scale 1 (1.16, [0.36; 1.95]) and mobility VAP Scale 2 (0.86, [0.04; 1.67]), resulting in a decrease over time. Further details are shown in Table 3.

**Fig. 2:**
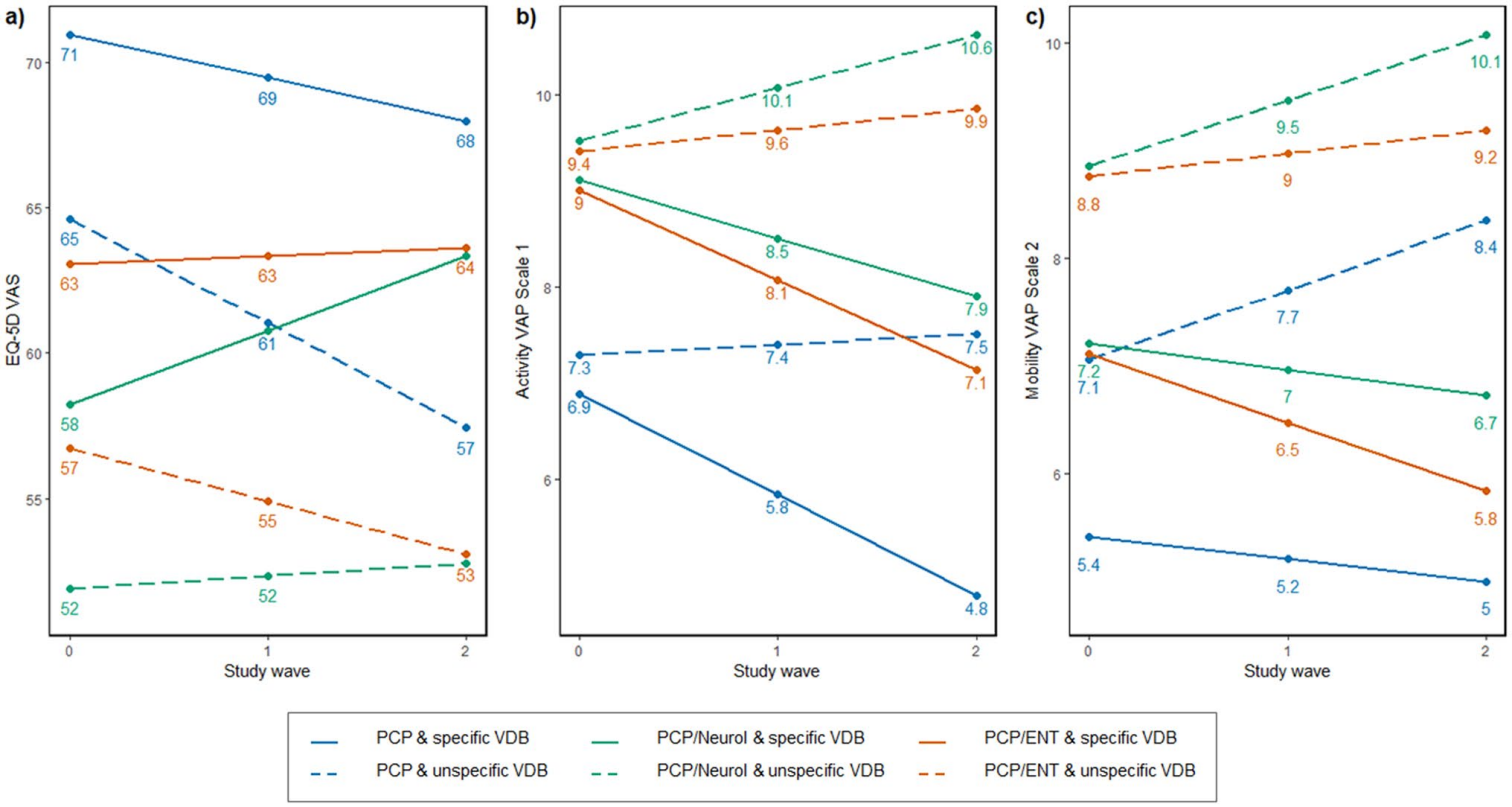
Difference in the predicted values of an exemplary patient between the distinct combination of referral cluster and diagnosis of VDB for **a** health-related quality of life measured by the visual analogue scale of the EQ-5D and functioning measured by the **b** activity scale and **c** mobility scale of the vestibular activities and participation questionnaire. The *numbers* show the predicted value at each time point

Figure 2 displays the differences in the predicted values between the distinct combinations of clusters and diagnosis of VDB for HRQoL and functioning over time for a fictional person based on the longitudinal linear mixed models. The predicted values apply for a 78-year-old exemplary female patient, living in Bavaria with no comorbidities related to a neurologist or ENT-specialist and no graduation or lower secondary education 1.

## Discussion

This is one of the first studies to systematically analyze referral trajectories of older patients with vertigo, dizziness and balance problems (VDB) in primary care. In our study, we identified three referral patterns using state sequence analysis (SSA).

Primary care physician (PCP) only without further referral, PCP and neurologist, and PCP and ENT specialist. Comorbidities and regional health care characteristics determined these typical referral patterns. Referral patterns and specificity of diagnosis were predictors of patient’s health-related quality of life (HRQoL). Patients with an unspecific diagnosis of VDB were at risk of reduced HRQoL and limited functioning.

It is not surprising that patients in our study were most frequently managed solely by the PCP without further referral to a neurologist or an ENT specialist, which confirms earlier findings from the literature [22, 38, 39].

Specific VDB diagnoses were not an indicator for referral to a specialist in our study. Arguably, VDB in older adults is seen as a health problem that can be managed in primary care. While most VDB diagnoses can be managed by the PCP, as proposed by the German DEGAM-Guideline (S3) for the treatment of VDB in the primary care setting [40], our earlier work indicated that PCPs report considerable uncertainty and lack of routine in VDB diagnosis and treatment [11]. Thus, absence of referral to the specialist partially undermines the logic that referral is needed in certain cases of VDB to make use of the specialist’s expertise for the respective disease [9, 10]. In contrast, VDB patients in our study with a neurological comorbidity (such as multiple sclerosis, Parkinson’s disease, and epilepsy) indeed were more often referred to neurologists, suggesting that these health conditions were seen as severe enough to elicit a referral.

Interestingly, the referral patterns greatly differed between the two federal states with patients from Saxony being less likely to be referred to specialist care. It has to be noted that Saxony is one of the eastern federal states of the former German Democratic Republic (GDR). Health system in the GDR was largely based on public ambulatory PCP clinics. Our results thus imply that in a situation, where getting the correct diagnosis and efficient treatment is a great challenge [3, 4], referral patterns in patients with VDB are influenced by the surrounding health care characteristics, as has also been reported for other indications [12, 14, 15].

Our study found evidence that patient reported HRQoL was affected by referral trajectories. Patients that consulted both the PCP and a neurologist had a significantly lower HRQoL at the beginning of the study, but did improve over time, approaching the HRQoL of the other patients. This might reflect the specific referral process where a neurologist was able to contribute to the effective management of the underlying neurological condition. However, our results also indicate that this potentially effective management of VDB did not affect VDB-specific functioning. This is in line with earlier studies showing that adequate management of VDB is a challenge [3].

In our study, patients with an unspecific diagnosis of VDB, i.e., cases in which the specific cause of VDB remained unspecified, were significantly at risk of unfavorable development of functioning. It has been mentioned repeatedly that VDB in older patients can have multiple causes [5, 6] and often expresses itself in ambiguous symptoms, resulting in unspecific diagnoses [41] and therefore unspecific and potentially ineffective treatment [42]. It has been shown that PCPs tend to abstain from referral of patients with symptoms that are either ambiguous or unfamiliar [38].

We are aware that our study has some limitations. Information on referrals in our study was based on self-report. Whether a reported consultation was related to VDB and whether the patients were actually referred to the specialists by the PCP was not assessed. Chances are given that patients did consult a specialist without having been referred by the PCP, which is possible in the German health care system. However, we are confident that this is only the minority of cases as the patients do have an acute episode of VDB and referrals to neurologists and ENT-specialists are rather common in this group of patients [22]. The VDB diagnoses used in this analysis were solely based on the assessments of the participating PCPs and thus might be partially inaccurate, since PCPs reported difficulties in establishing an accurate VDB diagnosis in the past [11] therefore frequently over-diagnosing unspecific VDB in patients who later were diagnosed with a specific cause of VDB [42]. The SSA used in this study is of an exploratory nature. An average silhouette width of 0.44 and a Hubert’s C index of 0.08, which were used as quality indicators for the clustering, indicate that the clustering structure identified has to be considered weak, yet existent. Referral trajectories in this study consist of three waves and do not allow any statements for longer than 1 year. This is especially important as we are not able to predict whether the trend for the different development of HRQoL between the referral patterns and for the different development of functioning between the patients with a specific and patients with an unspecific diagnosis, which we found in our analysis, continues. We therefore strongly suggest to further review our findings, including higher case numbers and a longer follow-up period.

In conclusion, current referral trajectories in a primary care setting in older patients with VDB were determined by present comorbidities of the patients and the regional healthcare characteristics. Referral patterns affected patients’ HRQoL. Although our analysis was of exploratory nature it indicates that unspecific VDB diagnoses increase the risk of ineffective management and consequently impaired functioning. Implementation of evidence-based standardized care pathways for management and referral of patients with VDB might be one potential solution to this problem.

## Supplementary Information

Below is the link to the electronic supplementary material.

Supplementary file1 (DOCX 14 KB)

Supplementary file2 (DOCX 32 KB)

Supplementary file3 (DOCX 18 KB)

Supplementary file4 (DOCX 467 KB)
