# Supplementary material for: Referral trajectories in patients with vertigo, dizziness and balance disorders and their impact on health-related quality of life and functioning: results from the longitudinal multicenter study MobilE-TRA
Source: J Neurol. 2022 Mar 30;269(12):6211–21. doi: 10.1007/s00415-022-11060-8 (PMC9618552; doi:10.1007/s00415-022-11060-8)
Supplement: Supplementary file 1 — Supplementary file1 (DOCX 14 KB) [file 415_2022_11060_MOESM1_ESM.docx]

**Referral trajectories in patients with vertigo, dizziness and balance disorders and their impact on health-related quality of life and functioning – Results from the longitudinal multicenter study** **MobilE-TRA, Journal of Neurology**

Benedict Katzenberger^1,5,§^, Daniela Koller^1,5^, Ralf Strobl^1,4^, Rebecca Kisch^1^, Linda Sanftenberg^2^, Karen Voigt^3^, Eva Grill^1, 4^

^1^ Institute for Medical Information Processing, Biometry and Epidemiology, Ludwig-Maximilians-Universität München, Munich, Germany
^2^ Institute of General Practice and Family Medicine, University Hospital, Ludwig-Maximilians-Universität München, Munich, Germany
^3^ Department of General Practice/Medical Clinic III, Faculty of Medicine, Technische Universität Dresden, Dresden, Germany
^4^ German Center for Vertigo and Balance Disorders, University Hospital, Ludwig-Maximilians-Universität München, Munich, Germany
^5^ Munich Center of Health Sciences, Ludwig-Maximilians-Universität München, Munich, Germany

^§^ Corresponding author

Benedict Katzenberger, M.Sc. Public Health

Institute for Medical Information Processing, Biometrics and Epidemiology,

Ludwig-Maximilians-Universität München, Marchioninistraße 15, 81377 Munich, Germany

Phone.: + 49 89 4400 77373

E-mail: Benedict.Katzenberger@med.uni-muenchen.de

Supplementary material 1: ICD-10 codes of included patients and related diagnoses

| ICD-10 code | VDB diagnosis |
| --- | --- |
| R42 | Dizziness and giddiness |
| A88.1 | Epidemic vertigo |
| E53.8 | Deficiency of other specified B group vitamins |
| F45.8 | Other somatoform disorders |
| G11.8 | Other hereditary ataxias |
| G43.1 | Other headache syndromes |
| G45.0 | Vertebro-basilar artery syndrome |
| G62 | Other polyneuropathies |
| G63 | Polyneuropathy in diseases classified elsewhere |
| H55 | Nystagmus and other irregular eye movements |
| H83.0–2 | Other diseases of inner ear |
| I95.1 | Orthostatic hypotension |
| N95.1 | Menopausal and female climacteric states |

*ICD-10, International classification of diseases, 10th Revision*
*VDB = Vertigo, dizziness and balance problems*
