# Supplementary material for: Referral trajectories in patients with vertigo, dizziness and balance disorders and their impact on health-related quality of life and functioning: results from the longitudinal multicenter study MobilE-TRA
Source: J Neurol. 2022 Mar 30;269(12):6211–21. doi: 10.1007/s00415-022-11060-8 (PMC9618552; doi:10.1007/s00415-022-11060-8)
Supplement: Supplementary file 2 — Supplementary file2 (DOCX 32 KB) [file 415_2022_11060_MOESM2_ESM.docx]

**Referral trajectories in patients with vertigo, dizziness and balance disorders and their impact on health-related quality of life and functioning – Results from the longitudinal multicenter study** **MobilE-TRA, Journal of Neurology**

Benedict Katzenberger^1,5,§^, Daniela Koller^1,5^, Ralf Strobl^1,4^, Rebecca Kisch^1^, Linda Sanftenberg^2^, Karen Voigt^3^, Eva Grill^1, 4^

^1^ Institute for Medical Information Processing, Biometry and Epidemiology, Ludwig-Maximilians-Universität München, Munich, Germany
^2^ Institute of General Practice and Family Medicine, University Hospital, Ludwig-Maximilians-Universität München, Munich, Germany
^3^ Department of General Practice/Medical Clinic III, Faculty of Medicine, Technische Universität Dresden, Dresden, Germany
^4^ German Center for Vertigo and Balance Disorders, University Hospital, Ludwig-Maximilians-Universität München, Munich, Germany
^5^ Munich Center of Health Sciences, Ludwig-Maximilians-Universität München, Munich, Germany

^§^ Corresponding author

Benedict Katzenberger, M.Sc. Public Health

Institute for Medical Information Processing, Biometrics and Epidemiology,

Ludwig-Maximilians-Universität München, Marchioninistraße 15, 81377 Munich, Germany

Phone.: + 49 89 4400 77373

E-mail: Benedict.Katzenberger@med.uni-muenchen.de

Supplementary material 2: Flow chart of patient recruitment in MobilE-TRA and inclusion to this analysis

For the baseline survey of MobilE-TRA, 19 practices (seven from Munich, 12 from Dresden) successfully recruited 258 eligible patients (111 from Dresden and 147 from Munich) for study participation.

For the first follow-up, 257 patients were re-contacted (one patient had died before the first follow-up). In total, 240 participated in follow-up one, five denied further participation, 12 did not reply despite a reminder. The resulting follow-up one response rate was 93.4% of all baseline participants. Out of the 240 follow-up one participants, three returned their questionnaire but simultaneously refused to be contacted for follow-up two. Thus, 248 patients were re-contacted for follow-up two. In total, 231 participated in follow-up two, resulting in a follow-up two response of 93.1%.

A total of 158 patients of the 258 eligible patients from the baseline survey suffered from VDB. There was no lost to follow-up in this patient group. Patients with VDB were included in this analysis, if they provided valid information on consulted physicians during the baseline assessment and at least one follow-up assessment.

Participants follow-up 2

n=231

Participants baseline

n=258

Participants follow-up 1 n=240

Contacted follow-up 1 n=257

Contacted follow-up 2 n=248

PCPs participating

n=19

Follow-up 1 missing

n=11

Non-responder questionnaire

n=6

Participants baseline with VDB

n = 158

**Valid Information on
physicians consulted**

**n = 141**

No lost to follow-up

**Fig A1** Flow chart of patient recruitment in MobilE-TRA and inclusion to this analysis
