# Supplementary material for: Referral trajectories in patients with vertigo, dizziness and balance disorders and their impact on health-related quality of life and functioning: results from the longitudinal multicenter study MobilE-TRA
Source: J Neurol. 2022 Mar 30;269(12):6211–21. doi: 10.1007/s00415-022-11060-8 (PMC9618552; doi:10.1007/s00415-022-11060-8)
Supplement: Supplementary file 3 — Supplementary file3 (DOCX 18 KB) [file 415_2022_11060_MOESM3_ESM.docx]

**Referral trajectories in patients with vertigo, dizziness and balance disorders and their impact on health-related quality of life and functioning – Results from the longitudinal multicenter study** **MobilE-TRA, Journal of Neurology**

Benedict Katzenberger^1,5,§^, Daniela Koller^1,5^, Ralf Strobl^1,4^, Rebecca Kisch^1^, Linda Sanftenberg^2^, Karen Voigt^3^, Eva Grill^1, 4^

^1^ Institute for Medical Information Processing, Biometry and Epidemiology, Ludwig-Maximilians-Universität München, Munich, Germany
^2^ Institute of General Practice and Family Medicine, University Hospital, Ludwig-Maximilians-Universität München, Munich, Germany
^3^ Department of General Practice/Medical Clinic III, Faculty of Medicine, Technische Universität Dresden, Dresden, Germany
^4^ German Center for Vertigo and Balance Disorders, University Hospital, Ludwig-Maximilians-Universität München, Munich, Germany
^5^ Munich Center of Health Sciences, Ludwig-Maximilians-Universität München, Munich, Germany

^§^ Corresponding author

Benedict Katzenberger, M.Sc. Public Health

Institute for Medical Information Processing, Biometrics and Epidemiology,

Ludwig-Maximilians-Universität München, Marchioninistraße 15, 81377 Munich, Germany

Phone.: + 49 89 4400 77373

E-mail: Benedict.Katzenberger@med.uni-muenchen.de

Supplementary material 3: Assessment of comorbidities in MobilE-TRA

The assessment of the comorbidities was accomplished by asking the patient’s primary care physician (PCP) about the present comorbidities during the baseline assessment. Comorbidities were reported by the PCP using the Charlson Comorbidity Index [24]. Following recommendations [25], we added further comorbidities to the index list that had shown to be of high relevance in older adult populations and potentially might influence HRQoL [26]. The PCPs were asked about the presence of the following comorbidities:

- pulmonary diseases, such as asthma, emphysema, and chronic bronchitis
- Inflammatory joint disease (e.g. arthritis) or rheumatism
- Cancer and other malignant tumor diseases
- diabetes mellitus
- gastrointestinal disease (e.g. stomach or duodenal ulcer, colon inflammation, cholecystitis)
- heart disease (e.g. angina pectoris, cardiac insufficiency, coronary heart disease)
- stroke
- other neurologic disease (e.g. multiple sclerosis, Parkinson’s disease, epilepsy)
- kidney disease
- liver disease (e.g. cirrhosis)
- eye diseases (excluding hyperopia and myopia)
- hypertension
- further comorbidities not mentioned above
