# Supplementary material for: Referral trajectories in patients with vertigo, dizziness and balance disorders and their impact on health-related quality of life and functioning: results from the longitudinal multicenter study MobilE-TRA
Source: J Neurol. 2022 Mar 30;269(12):6211–21. doi: 10.1007/s00415-022-11060-8 (PMC9618552; doi:10.1007/s00415-022-11060-8)
Supplement: Supplementary file 4 — Supplementary file4 (DOCX 467 KB) [file 415_2022_11060_MOESM4_ESM.docx]

**Referral trajectories in patients with vertigo, dizziness and balance disorders and their impact on health-related quality of life and functioning – Results from the longitudinal multicenter study** **MobilE-TRA, Journal of Neurology**

Benedict Katzenberger^1,5,§^, Daniela Koller^1,5^, Ralf Strobl^1,4^, Rebecca Kisch^1^, Linda Sanftenberg^2^, Karen Voigt^3^, Eva Grill^1, 4^

^1^ Institute for Medical Information Processing, Biometry and Epidemiology, Ludwig-Maximilians-Universität München, Munich, Germany
^2^ Institute of General Practice and Family Medicine, University Hospital, Ludwig-Maximilians-Universität München, Munich, Germany
^3^ Department of General Practice/Medical Clinic III, Faculty of Medicine, Technische Universität Dresden, Dresden, Germany
^4^ German Center for Vertigo and Balance Disorders, University Hospital, Ludwig-Maximilians-Universität München, Munich, Germany
^5^ Munich Center of Health Sciences, Ludwig-Maximilians-Universität München, Munich, Germany

^§^ Corresponding author

Benedict Katzenberger, M.Sc. Public Health

Institute for Medical Information Processing, Biometrics and Epidemiology,

Ludwig-Maximilians-Universität München, Marchioninistraße 15, 81377 Munich, Germany

Phone.: + 49 89 4400 77373

E-mail: Benedict.Katzenberger@med.uni-muenchen.de

Supplementary material 4: Directed Acyclic Graphs

To avoid bias by over-adjustment or collider bias, we used directed acyclic graphs (DAGs) in order to arrive at a parsimonious set of variables, the minimal sufficient adjustment set, for estimating the effect of referral patterns on HRQoL and functioning.

**
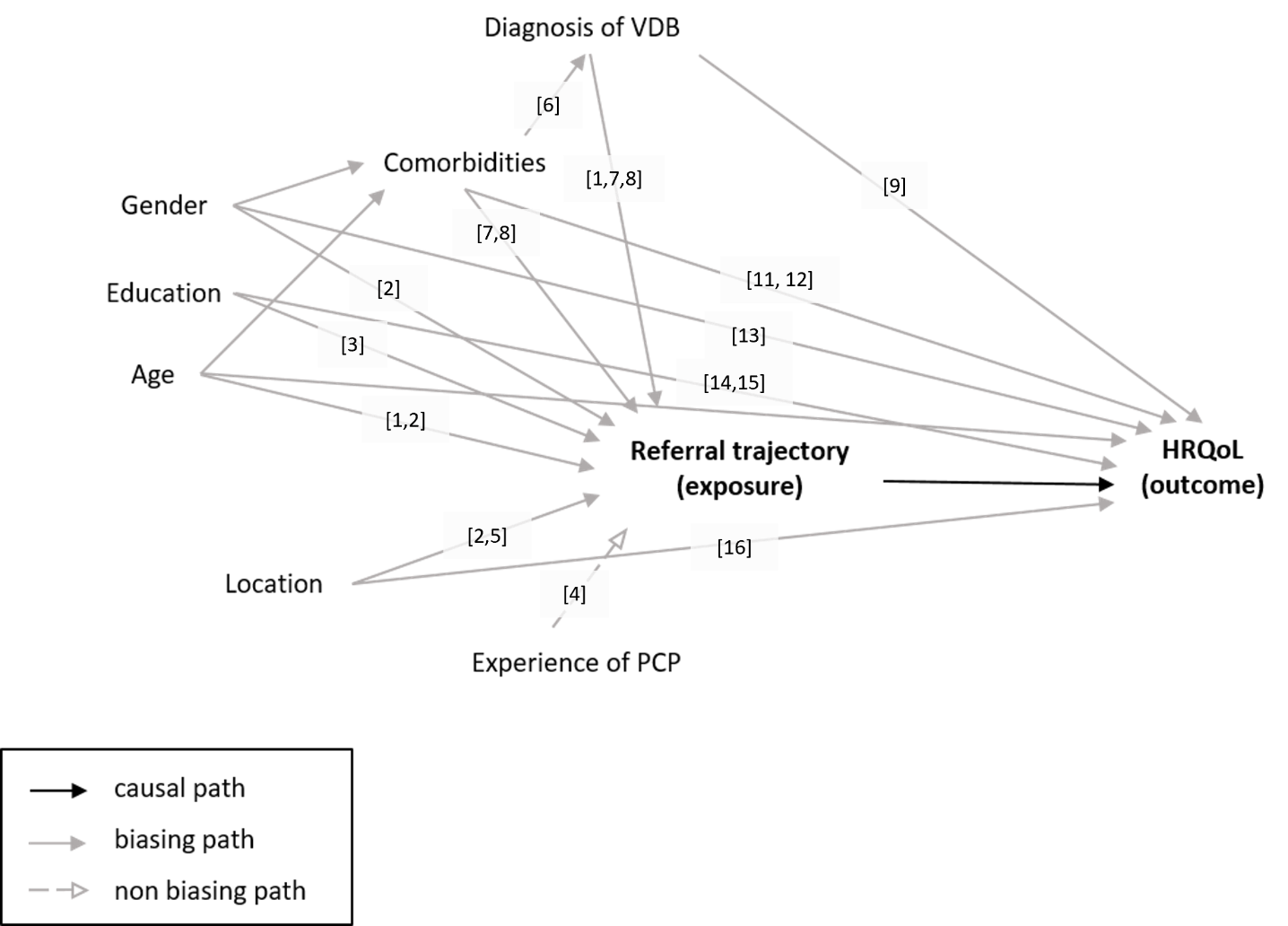
**

**Fig A2** Directed acyclic graph for the effect of referral trajectories on health-related quality of life

Literature

| 1 | Stephan A-J, Kovacs E, Phillips A, Schelling J, Ulrich SM, Grill E (2018) Barriers and facilitators for the management of vertigo: a qualitative study with primary care providers. Implement Sci 13:1-10. http://dx.doi.org/10.1186/s13012-018-0716-y |
| --- | --- |
| 2 | Forrest CB, Nutting PA, Von Schrader S, Rohde C, Starfield B (2006) Primary care physician specialty referral decision making: patient, physician, and health care system determinants. Med Decis Making 26:76-85. https://doi.org/10.1177/0272989X05284110 |
| 3 | Fylkesnes K (1993) Determinants of health care utilization—visits and referrals. Scand J Soc Med 21:40-50. https://doi.org/10.1177/140349489302100107 |
| 4 | Sun Z, Ng C, Halkett G, Meng R, Jiwa M (2013) An investigation of factors that influence general practitioners' referral of computed tomography scans in patients with headache. Int J Clin Pract 67:682-690. http://dx.doi.org/10.1111/ijcp.12186 |
| 5 | Morgan M, Jenkins L, Ridsdale L (2007) Patient pressure for referral for headache: a qualitative study of GPs' referral behaviour. Brit J Gen Pract 57:29-35. |
| 6 | Maarsingh OR, Dros J, Schellevis FG, van Weert HC, Bindels PJ, van der Horst HE (2010) Dizziness reported by elderly patients in family practice: prevalence, incidence, and clinical characteristics. BMC Fam Pract 11:1-9. http://dx.doi.org/10.1186/1471-2296-11-2 |
| 7 | Bösner S, Träger S, Hirsch O, Becker A, Ilhan M, Baum E, Donner-Banzhoff N (2011) Vom Hausarzt zum Facharzt–aktuelle Daten zu Überweisungsverhalten und-motiven. Z Allg 87:371-377. http://dx.doi.org/10.3238/zfa.2011.037 |
| 8 | Gröber-Grätz D, Moßhammer D, Bölter R, Ose D, Joos S, Natanzon I (2011) Welche Kriterien beeinflussen Hausärzte bei der Überweisung zum Spezialisten in der ambulanten Versorgung? Eine qualitative Studie zur Sichtweise von Hausärzten. ZEFQ 105:446-451. http://dx.doi.org/10.1016/j.zefq.2011.06.001 |
| 9 | Ten Voorde M, Van Der Zaag-Loonen H, Van Leeuwen R (2012) Dizziness impairs health-related quality of life. Qual Life Res 21:961-966. http://dx.doi.org/10.1007/s11136-011-0001-x |
| 10 | Ciorba A, Bianchini C, Scanelli G, Pala M, Zurlo A, Aimoni C (2017) The impact of dizziness on quality-of-life in the elderly. Eur Arch Oto-Rhino-L 274:1245-1250. https://doi.org/10.1007 |
| 11 | Amtmann D, Bamer AM, Kim J, Chung H, Salem R (2018) People with multiple sclerosis report significantly worse symptoms and health related quality of life than the US general population as measured by PROMIS and NeuroQoL outcome measures. Disabil Health J 11:99-107. https://dx.doi.org/10.1016/j.dhjo.2017.04.008 |
| 12 | Schrag A, Jahanshahi M, Quinn N (2000) How does Parkinson's disease affect quality of life? A comparison with quality of life in the general population. Movement Disord 15:1112-1118. https://dx.doi.org/10.1002/1531-8257(200011)15:6<1112::aid-mds1008>3.0.co;2-a |
| 13 | Kirchengast S, Haslinger B (2008) Gender differences in health-related quality of life among healthy aged and old-aged Austrians: cross-sectional analysis. Gender Medicine 5:270-278. https://dx.doi.org/10.1016/j.genm.2008.07.001 |
| 14 | Robert SA, Cherepanov D, Palta M, Dunham NC, Feeny D, Fryback DG (2009) Socioeconomic status and age variations in health-related quality of life: results from the National Health Measurement Study. J Gerontol B 64:378-389. http://dx.doi.org/10.1093/geronb/gbp012 |
| 15 | Mielck A, Reitmeir P, Vogelmann M, Leidl R (2013) Impact of educational level on health-related quality of life (HRQL): results from Germany based on the EuroQol 5D (EQ-5D). Eur J Public Health 23:45-49. http://dx.doi.org/10.1093/eurpub/ckr206 |
| 16 | Tsai S-Y, Chi L-Y, Lee L-S, Chou P (2004) Health-related quality of life among urban, rural, and island community elderly in Taiwan. J Formos Med Assoc 103:196-204. |


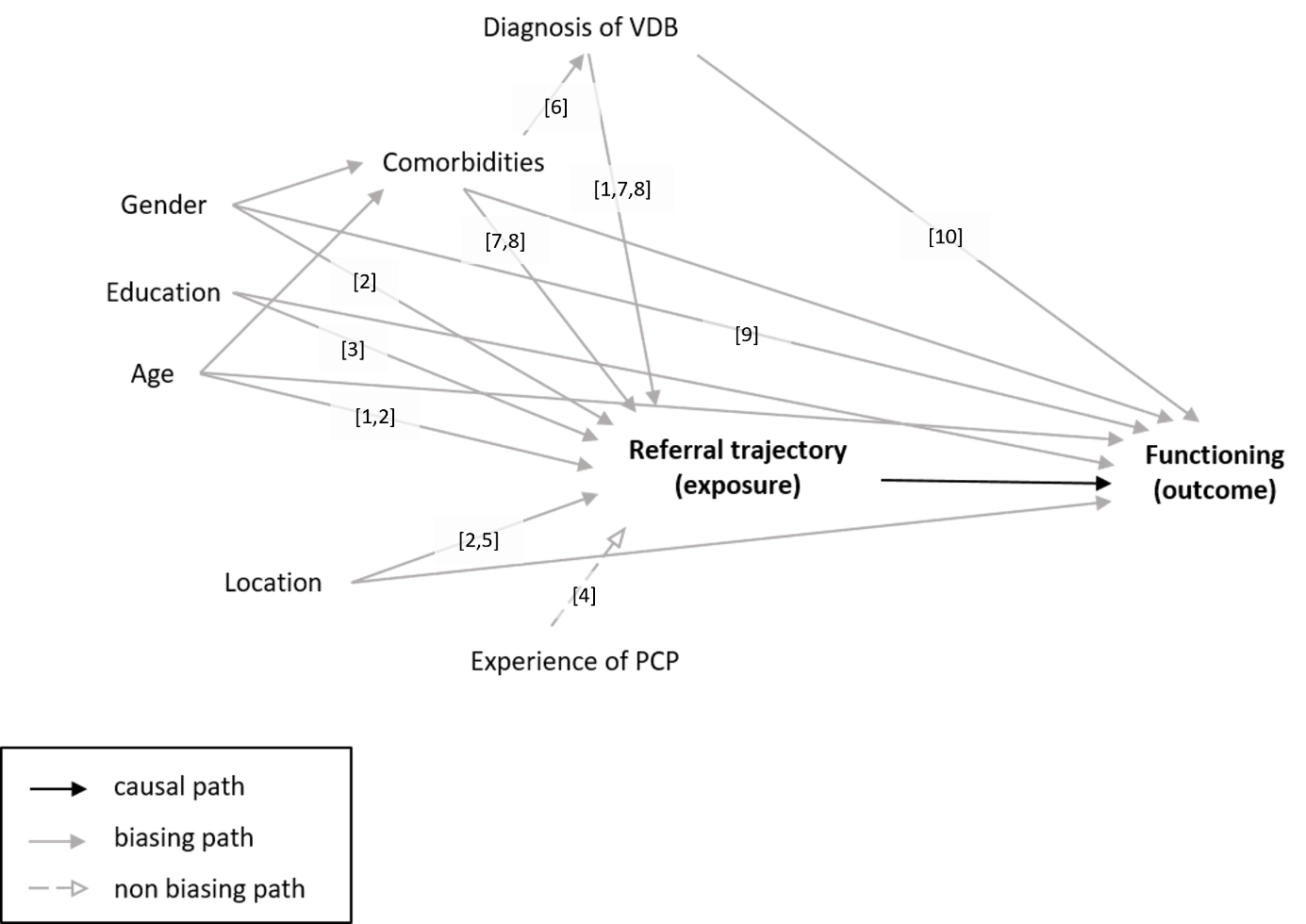


**Fig A3** Directed acyclic graph for the effect of referral trajectories on functioning

Literature

| 1 | Stephan A-J, Kovacs E, Phillips A, Schelling J, Ulrich SM, Grill E (2018) Barriers and facilitators for the management of vertigo: a qualitative study with primary care providers. Implement Sci 13:1-10. http://dx.doi.org/10.1186/s13012-018-0716-y |
| --- | --- |
| 2 | Forrest CB, Nutting PA, Von Schrader S, Rohde C, Starfield B (2006) Primary care physician specialty referral decision making: patient, physician, and health care system determinants. Med Decis Making 26:76-85. https://doi.org/10.1177/0272989X05284110 |
| 3 | Fylkesnes K (1993) Determinants of health care utilization—visits and referrals. Scand J Soc Med 21:40-50. https://doi.org/10.1177/140349489302100107 |
| 4 | Sun Z, Ng C, Halkett G, Meng R, Jiwa M (2013) An investigation of factors that influence general practitioners' referral of computed tomography scans in patients with headache. Int J Clin Pract 67:682-690. http://dx.doi.org/10.1111/ijcp.12186 |
| 5 | Morgan M, Jenkins L, Ridsdale L (2007) Patient pressure for referral for headache: a qualitative study of GPs' referral behaviour. Brit J Gen Pract 57:29-35. |
| 6 | Maarsingh OR, Dros J, Schellevis FG, van Weert HC, Bindels PJ, van der Horst HE (2010) Dizziness reported by elderly patients in family practice: prevalence, incidence, and clinical characteristics. BMC Fam Pract 11:1-9. http://dx.doi.org/10.1186/1471-2296-11-2 |
| 7 | Bösner S, Träger S, Hirsch O, Becker A, Ilhan M, Baum E, Donner-Banzhoff N (2011) Vom Hausarzt zum Facharzt–aktuelle Daten zu Überweisungsverhalten und-motiven. Z Allg 87:371-377. http://dx.doi.org/10.3238/zfa.2011.037 |
| 8 | Gröber-Grätz D, Moßhammer D, Bölter R, Ose D, Joos S, Natanzon I (2011) Welche Kriterien beeinflussen Hausärzte bei der Überweisung zum Spezialisten in der ambulanten Versorgung? Eine qualitative Studie zur Sichtweise von Hausärzten. ZEFQ 105:446-451. http://dx.doi.org/10.1016/j.zefq.2011.06.001 |
| 9 | Stephan A-J, Schwettmann L, Meisinger C, Ladwig K-H, Linkohr B, Thorand B, Schulz H, Peters A, Grill E (2021) Living longer but less healthy: The female disadvantage in health expectancy. Results from the KORA-Age study. Exp Gerontol 145:111196. http://dx.doi.org/10.1016/j.exger.2020.111196 |
| 10 | Obermann M, Bock E, Sabev N, Lehmann N, Weber R, Gerwig M, Frings M, Arweiler-Harbeck D, Lang S, Diener H-C (2015) Long-term outcome of vertigo and dizziness associated disorders following treatment in specialized tertiary care: the Dizziness and Vertigo Registry (DiVeR) Study. J Neurol 262:2083-2091. http://dx.doi.org/10.1007/s00415-015-7803-7 |
| 11 | Den Oudsten BL, Van Heck GL, De Vries J (2007) Quality of life and related concepts in Parkinson's disease: a systematic review. Movement Disord 22:1528-1537. http://dx.doi.org/10.1002/mds.21567 |
| 12 | Amtmann D, Bamer AM, Kim J, Chung H, Salem R (2018) People with multiple sclerosis report significantly worse symptoms and health related quality of life than the US general population as measured by PROMIS and NeuroQoL outcome measures. Disabil Health J 11:99-107. https://dx.doi.org/10.1016/j.dhjo.2017.04.008 |
| 13 | Brosel S, Strupp M (2019) The vestibular system and ageing. In: Harris J, Korolchuk V (eds) Biochemistry and Cell Biology of Ageing: Part II Clinical Science. Springer, Singapore, pp 195-225 |
| 14 | Ishiyama G (2009) Imbalance and vertigo: the aging human vestibular periphery. Semin Neurol 29:491-499. http://dx.doi.org/10.1055/s-0029-1241039 |
